# The effect of chronoradiotherapy on cervical cancer patients: A multicenter randomized controlled study

**DOI:** 10.3389/fonc.2022.1021453

**Published:** 2022-11-15

**Authors:** Ying Wang, Wan-Min Qiang, Jia-Qian Li, Ao-Mei Shen, Xiao-Cen Chen, Xiao-Fang Li, Bao-Zhong Zhang, Juan Xie, Rong Yan, Xiang-Hua Li, Zhao-Li Zhang, Cui-Ling Wang, Lai-You Li

**Affiliations:** ^1^ Nursing Department, Tianjin Medical University Cancer Institute & Hospital, National Clinical Research Center for Cancer, Tianjin, China; ^2^ Radiotherapy Department, Shaanxi Provincial Cancer Hospital, Xian, China; ^3^ Nursing Department, Shandong Cancer Hospital, Qingdao, China; ^4^ Nursing Department, Cangzhou People's Hospital, Cangzhou, China; ^5^ Nursing Department, Chongqing Cancer Hospital, Chongqing, China; ^6^ Nursing Department, Shanxi Provincial Cancer Hospital, Taiyuan, China; ^7^ Nursing Department, The Fourth Hospital of Hebei Medical University, Shijiazhuang, China

**Keywords:** radiotherapy, chronoradiotherapy, cervical cancer, radiation toxicity, radiotherapy effects

## Abstract

**Objectives:**

To investigate the short-term efficacy and radiotoxicity 3.543of chronoradiotherapy in patients with cervical cancer. We also examined the overall symptom score and quality of life (QOL) of patients who underwent morning radiotherapy and evening radiotherapy.

**Methods:**

We conducted a multicenter randomized controlled trial to compare the effects of morning radiotherapy (9:00–11:00 AM) with evening radiotherapy (7:00–9:00 PM) in cervical cancer patients receiving radiotherapy. From November 2021 to June 2022, 114 cervical cancer patients admitted to eight cancer center hospitals in Tianjin, Chongqing, Hubei, Shanxi, Shandong, Shaanxi, Hebei, and Cangzhou were randomly divided into the morning radiotherapy group (MG; N = 61) and the evening radiotherapy group (EG; N = 53). The short-term efficacy of radiotherapy on cervical cancer patients at different time points and the occurrence of radiotoxicity were explored after patients had undergone radiotherapy.

**Results:**

The total effective response (partial remission [PR] + complete remission [CR]) rate was similar across the two groups (93.5% vs. 96.3%, *p* > 0.05). However, the incidence of bone marrow suppression and intestinal reaction in the two groups were significantly different (*p* < 0.05). The patients in the MG had significantly higher Anderson symptom scores than patients in the EG (21.64 ± 7.916 vs. 18.53 ± 4.098, *p* < 0.05). In terms of physical activity, functional status, and overall QOL, the MG had significantly lower scores than the EG (*p* < 0.05). No other measures showed a significant difference between the groups.

**Conclusion:**

The radiotherapy effect of the MG was consistent with that of the EG. The incidence of radiation enteritis and radiation diarrhea in the MG was significantly higher than that in the EG; however, bone marrow suppression and blood toxicity in the EG were more serious than in the MG. Because of the small sample size of the study, we only examined the short-term efficacy of radiotherapy. Therefore, further clinical trials are needed to verify the efficacy and side effects of chronoradiotherapy.

**Clinical Trial Registration:**

http://www.chictr.org.cn/searchproj.aspx, Registration Number: ChiCTR2100047140.

## Introduction

A recent analysis revealed that cervical cancer remains a major threat to women. In 2020, there were an estimated 604,000 new cases of cervical cancer globally, which was the second most diagnosed cancer in women (1). Although cervical cancer is one of the leading causes of cancer-related death in women worldwide (2), nearly 90% of cervical cancer deaths occur in developing countries, with India and China accounting for 35% of the total cervical cancer burden (3).

Radiotherapy, alone or in combination with surgery or chemotherapy, is the main treatment for cervical cancer (4). Almost 80% of patients with cervical cancer undergo radiation therapy as part of their treatment (5). The aim of radiotherapy is to irradiate malignant tumors *via* ionizing radiation, and the cumulative effect of the irradiation dose destroys tumor cells (6). However, during the process of radiotherapy, although tumor cells are killed, the surrounding normal tissues are also damaged, which causes a series of toxic side effects.

Exposure to ionizing radiation during radiotherapy of the abdominopelvic region is associated with the development of treatment-limiting untoward symptoms. The consequences of damaging healthy cells can result in a series of adverse reactions ranging from acute radiation toxicity to organ damage and secondary cancers (7). Approximately 84% of patients undergo some form of acute radiation toxicity during radiation therapy for cervical cancer (7). The most common symptoms are hematological toxicity, gastrointestinal mucositis, diarrhea, nausea, and vomiting, which may lead to treatment interruptions, increased healthcare costs, and impaired quality of life (QOL) in patients undergoing irradiation. These adverse reactions are attributed to various factors, such as therapeutic, environmental, and genetic factors. In recent years, studies have explored how the time of day of radiotherapy administration affects radiation therapy outcomes to determine whether chrono-modulation may be beneficial (6–8).

The circadian rhythm is governed by an internal timing system that is regulated at the transcriptional level, creating networks of genes that oscillate on a 24-hour cycle (8). The cell cycle, proliferation, and cell death are closely intertwined with the circadian rhythm. Several recent studies have provided compelling evidence on the association between the circadian cycle and cancer; similar to healthy cells, tumor cells are rhythmic (9, 10), and their growth depends on circadian rhythms (11). It has been reported that each phase of the cell cycle corresponds to a different degree of radiosensitivity (12). Cells in or near mitosis (G2 and M phases) have the highest radiosensitivity, whereas cells in the S and G1 phases are less radiosensitive. Tumor cells also show time rhythms in metabolism and proliferation, which differ from those of healthy tissue cells. According to the different sensitivities of cells to radiation during different mitosis cycles, studies have investigated the time law of radiation sensitivity of tumor tissue and healthy tissue cells (12, 13). In line with circadian rhythm regularity, selecting a specific time to apply radiation therapy to tumors can significantly improve the efficacy of tumor radiation therapy (12, 13).

Chronoradiotherapy involves selecting the optimal radiotherapy time according to the body’s rhythm changes. It is aimed at protecting normal tissues as much as possible while killing tumor cells to the greatest extent to attenuate toxicity and increase efficiency. Radiotherapy can achieve a good curative effect, but the dose is roughly the maximum that the body can tolerate, which significantly limits the treatment of tumors. Therefore, ways in which to further improve the curative effect and minimize radiotoxicity is an important topic that requires urgent study. In this study, we investigated the radiation effects, radiotoxicity, and QOL in inoperable cervical cancer patients irradiated at different times of the day. Although chronoradiotherapy may be offered to cervical cancer patients as a new method, its efficacy and toxicity must be established. Current prospective randomized clinical data are lacking, and the use of chronoradiotherapy for the treatment of cervical carcinoma has not yet been established. Therefore, we conducted a multicenter prospective randomized study to assess the effectiveness of chronoradiotherapy in cervical cancer patients and to explore the relationship between the severity of acute gastrointestinal mucositis and the time of radiation in patients with carcinoma of the cervix.

## Materials and methods

### Study design

This was a multicenter randomized controlled trial (RCT) comparing morning radiotherapy (9:00–11:00 AM) with evening radiotherapy (7:00–9:00 PM) for cervical cancer patients undergoing radiotherapy. The study was registered with the Chinese Clinical Trial Registry (ref. Chi-CTR-2100047140) and was conducted from November 2021 to June 2022 at eight cancer center hospitals in the cities and provinces of Tianjin, Chongqing, Hubei, Shanxi, Shandong, Shaanxi, and Hebei. The Tianjin Medical University Cancer Institute and the Hospital Institutional Review Board approved the study protocol (approval number: bc2020185), and all caregivers provided informed consent. A total of 114 patients were registered during this period and were included in the study.

### Study participants

Patients were eligible if they fulfilled the inclusion and exclusion criteria. The inclusion criteria were as follows: 1) aged between 18 and 65 years; 2) cervical cancer patients with Federation International of Gynecology and Obstetrics (FIGO) stage IIB-IVA tumors confirmed by pathological biopsy to be nonmetastatic cervical cell carcinoma (see **Table 1** for details) (14); 3) a Karnofsky Performance Status (KPS) score of ≥ 70 points; 4) patients participated voluntarily and provided written informed consent.

**Table 1:** 2018 Federation International of Gynecology and Obstetrics (FIGO) Staging System for uterine cervical cancer.

Stage	Description
I	Carcinoma is strictly confined to the cervix (extension to the uterine corpus should be disregarded)
IA	Invasive carcinoma that can be diagnosed only with microscopy, with maximum depth of invasion < 5 mm
IA1	Stromal invasion < 3 mm in depth
IA2	Stromal invasion ≥ 3 mm and < 5 mm in depth
IB	Invasive carcinoma confined to the uterine cervix, with measured deepest invasion ≥ 5 mm
IB1*	Tumor measures < 2 cm in greatest dimension
IB2*	Tumor measures ≥ 2 cm and < 4 cm in greatest dimension
IB3*	Tumor measures ≥ 4 cm in greatest dimension
II	Carcinoma invades beyond the uterus but has not extended onto the lower third of the vagina or to the pelvic wall
IIA	Limited to the upper two-thirds of the vagina without parametrial involvement
IIA1	Tumor measures < 4 cm in greatest dimension
IIA2	Tumor measures ≥ 4 cm in greatest dimension
IIB	With parametrial involvement but not up to the pelvic wall
III	Carcinoma involves the lower third of the vagina and/or extends to the pelvic wall and/or causes hydronephrosis or nonfunctioning kidney and/or involves pelvic and/or para-aortic lymph nodes
IIIA	Involves the lower third of the vagina, with no extension to the pelvic wall
IIIB	Extension to the pelvic wall and/or hydronephrosis or nonfunctioning kidney from tumor
IIIC*	Involvement of pelvic and/or para-aortic lymph nodes, irrespective of tumor size and extent†
IIIC1*	Pelvic lymph node metastasis only
IIIC2*	Para-aortic lymph node metastasis
IV	Carcinoma has extended beyond the true pelvis or has involved (biopsy-proven) the mucosa of the bladder or rectum
IVA	Spread to adjacent pelvic organs
IVB	Spread to distant organs

Pathologic analysis, where available, can be used to supplement clinical findings for all stages. FIGO, International Federation of Gynecology and Obstetrics (adapted, under a CC BY license, from reference 1)

*New stages from the 2009 FIGO system.

†Stage IIIC should be annotated with r (radiology) or p (pathologic analysis) to indicate the method used to allocate this stage. Imaging modality or pathologic technique should also be documented.

The exclusion criteria were as follows: 1) clinically significant diseases (e.g., second primary tumor, severe infection, acute and chronic intestinal diseases or hemorrhoids, mental diseases, and systemic immune diseases) that might interfere with the primary endpoint assessment; 2) patients who had undergone major surgery within the 14 days before enrollment; 3) patients with serious liver, kidney, or another organ dysfunction.

### Randomization, allocation concealment, and blinding

Randomization was performed before the beginning of the intervention using a random number table technique to ensure an equal number of participants in each group. The random allocation sequence was produced using the Statistical Analysis Software (SAS), version 9.4 (SAS Institute, Inc., Cary, NC, USA). Eight sets of random sequences with a sample size of 114 cases were generated by a computer, randomly grouped in a 1:1 ratio, and each center was divided into two sets of random sequences. After the participants provided informed consent and underwent baseline assessments, they were randomly assigned to receive either morning radiotherapy (i.e., the morning radiotherapy group [MG]) or evening radiotherapy (i.e., the evening radiotherapy group [EG]). Allocation concealment was assured by using sequentially numbered, opaque, sealed, and stapled envelopes that were distributed to the participants by the project manager. To avoid the disclosure of group assignment, aluminum foil was used to keep the envelope invisible, even under intense light. The group assignment (intervention or control group) was replaced by group A or B, so that the research assistant who collected and entered the study data into a database remained blinded to group allocation throughout the study.

### Radiotherapy regimen

Both the MG and EG were treated with a uniform treatment combining external beam irradiation and high dose-rate (HDR) brachytherapy, without low dose-rate (LDR) brachytherapy. The external irradiation area has a large area to primarily address the problem of lymph node metastasis in the abdomen and pelvis. We used high-energy 6 MV and above X-rays for irradiation, and the irradiation dose was (50.4 Gy, 5–6 weeks, 28 fractions). HDR brachytherapy with iridium 192 HDR at a dose rate of 12–70/h, was initiated when the external radiation dose reached 30 Gy, and short-range radiation was added (30 Gy, five fractions). The samples in the MG received radiotherapy from 9:00 to 11:00 AM, whereas those in the EG received radiotherapy from 7:00 to 9:00 PM. In addition to radiotherapy, patients received a cisplatin chemotherapy regimen, (25 mg/m2 intravenously Guttae for 4–6 weeks). All the samples of this study in both groups received chemotherapy over the same time period, which was from 9:00 to 11:00 AM. We assessed the QOL of samples at baseline and the end of treatment. In addition, patients recorded any complaints of discomfort in a booklet we developed during this period to improve the compliance rate of patients.

### Interventions

Before enrollment, patients in both groups received unified dietary guidance and radiotherapy-related health education. One day before radiotherapy, patients underwent blood tests, imaging examinations, such as chest X-ray, pelvic ultrasound, and electrocardiogram, and other baseline assessments. During radiation therapy, blood tests and radiotoxicity were assessed weekly by a trained observer blinded to group assignment. In addition, during radiotherapy, patients were instructed to use a uniform douche for vaginal douches twice per week. For radiotherapy-related symptoms, such as diarrhea and enteritis, we provided standardized treatments in strict accordance with the requirements of the protocol and maintained a complete record of the course of treatments. Finally, patients were evaluated for efficacy on the day following the final radiotherapy session (i.e., the day after the final brachytherapy).

### Outcome measures

All outcomes were measured at baseline before the treatment and at the end of treatment. The curative effect of radiotherapy was evaluated according to the Response Evaluation Criteria in Solid Tumors (RECIST) 1.1 (see **Table 2** for details) (15). Toxicity was assessed using the Radiation Therapy Oncology Group’s common toxicity criteria (16). Myelosuppression was assessed using the myelosuppression grading of the World Health Organization. The Functional Assessment of Cancer Therapy-Cervix scale (17) was used to assess the QOL of patients during radiotherapy. Other radiation-related adverse reactions, such as pain, vomiting, sadness, and insomnia, were assessed using the M.D. Anderson Symptom Inventory (MDASI) (18). The case collection period was from August 2021 to December 2021. A trained observer assessed the results of patient assessments and completed unified case report forms, which included general information and relevant assessment results if patients visited the hospital for surveillance as an outpatient.

**Table 2:** Response Evaluation Criteria in Solid Tumors (RECIST 1.1).

Grade	Efficacy evaluation criteria
CR	Disappearance of all pleural and non-pleural disease (including pleural thickening considered to represent tumor).
PR	Summed measurement decrease by at least 30% from the baseline scan summed measurement, which must be confirmed at a subsequent follow-up scan at least 4 weeks later (at which time the summed measurement must not exceed 70% of the baseline scan summed measurement).
SD	Summed measurement increase by at least 20% from the nadir of the summed measurements from all prior scans (up to and including the baseline scan), even if the summed measurement is < 70% of the baseline scan summed measurement; classification as PD also requires an absolute summed measurement increase of at least 5 mm over the nadir summed measurement. An unequivocal new non-pleural lesion or an unequivocal new focus of pleural thickening that exceeds the minimum measurable size (and represents either a pleural tumor mass physically distinct from that associated with existing measurement sites or a region of a previously existing pleural tumor mass that would now unequivocally qualify as a measurement site) would be considered progressive disease.
PD	A decrease in the summed measurement that does not qualify as PR, or an increase in the summed measurement that does not qualify as PD.

CR, complete remission; PR, partial remission; SD, stable disease; PD, progression of disease.

### Sample size calculation

Before conducting the study, we calculated the approximate sample size considering the incidence of diarrhea in the two groups of chronoradiotherapy in relevant literature using the Power Analysis and Sample Size software version 15.0 (NCSS, Inc., USA). sample size calculation software: MG: 87.39%, EG: 68.18%, β = 0.1, test efficiency 1 – β = 80%, α = 0.05, N1 = N2 = 47, a total of 94 cases. Accounting for a 10% dropout rate, we determined that a minimum sample size of 104 cases would be required with N1 = N2 = 52, respectively.

### Statistical analysis

The data were analyzed using the Statistical Product and Service Solution version 21.0 (IBM Institute, Inc., Stanford, CA, USA). The count data are expressed as frequencies and percentages. Data that conformed to a normal distribution are described as means ± standard deviations, and those that were not normally distributed are described as medians and interquartile ranges. Baseline characteristics in the control and intervention groups were analyzed to assess whether there were between-group differences. To assess differences in mean scores between the intervention and control groups, we used a parametric test (t-test) for scores with a normal distribution and a non-parametric test (Mann–Whitney U test) for scores with non-normal distribution. Chi-square analysis was used to compare differences between the two groups after radiotherapy. Statistical significance was defined as a two-sided *p* < 0.05. Excel (Microsoft Office Home and Student 2019) was used for the analysis.

## Results

### Patients

**Figure 1:**
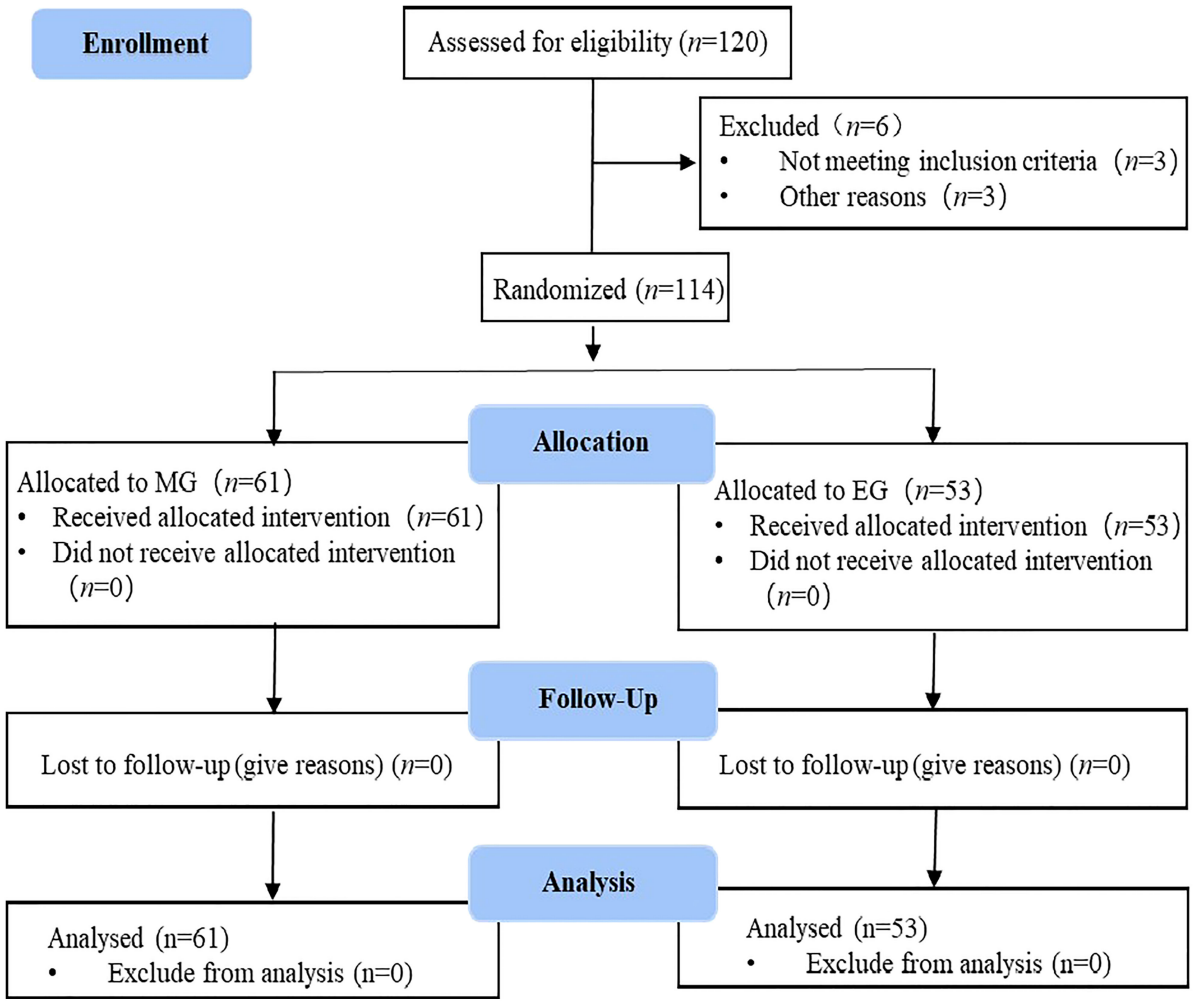
The CONSORT chart of the study.

Between November 2021 and June 2022, this study initially included 120 patients. However, three cases were excluded because they did not meet the inclusion criteria, and three cases dropped out of the study before the follow-up. Finally, 114 cases were included, which comprised 61 patients in the MG and 53 patients in the EG (**Figure 1**). All patients were pathologically diagnosed with cervical cancer with clinical stage IIB-IVA and had no indication for surgery. All the samples in this study had the same circadian rhythm sleeping at night and doing daily activities on daytime. Before the intervention, there were no statistical differences between the groups in terms of baseline characteristics, including general demographic, disease, or social data (*p* < 0.05). The baseline characteristics of MG and EG are shown in **Table 3**.

**Table 3:** Baseline characteristics of patients.

Characteristic	Morning	Evening	*t/x^2^*	*P*
	Group (*n* = 61)	Group (*n* = 53)		
Age (years)	56.85 ± 9.80	55.72 ± 9.55	0.625^a^	0.533
Weight (kg)	62.47 ± 12.00	62.77 ± 8.65	0.154^a^	0.427
Height (cm)	156.11 ± 19.03	160.26 ± 5.30	1.535^a^	0.193
Body surface area (m^2^)	1.61 ± 0.14	1.64 ± 0.11	1.19^a^	0.072
Blood pressure (mmHg)	94.23 ± 12.19	92.74 ± 12.26	0.651^a^	0.519
Body temperature (°C)	36.30 ± 0.20	36.29 ± 0.22	0.194^a^	0.303
Pulse (time)	79.72 ± 10.08	79.55 ± 8.82	0.097^a^	0.062
Breath	18.87 ± 1.638	18.58 ± 1.865	0.866^a^	0.329
Job category				
Retired	9 (14.8)	5 (9.4)	3.543^b^	0.471
Staff	9 (14.8)	6 (11.3)		
Peasant	19 (31.1)	23 (43.4)		
Freelancer	3 (4.9)	5 (9.4)		
Unemployed	21 (34.4)	14 (26.4)		
Educational level				
Primary and below	24 (39.3)	17 (32.1)	4.019^b^	0.547
Junior high school	26 (42.6)	20 (37.7)		
High school	5 (8.2)	11 (20.8)		
Technical secondary school	2 (3.3)	1 (1.9)		
College				
Junior college	3 (4.9)	3 (5.7)		
Bachelor’s degree or above	1 (1.6)	1 (1.8)		
Marital status				
Unmarried	1 (1.6)	0 (0)	2.020^b^	0.364
Married	60 (98.4)	52 (98.1)		
Remarried	0 (0)	1 (1.9)		
Divorced	0 (0)	0 (0)		
Other	0 (0)	0 (0)		
Per capita monthly household income (yuan)				
< 3000				
3000–5000	14 (23.0)	8 (15.1)	2.289^b^	0.515
5001–10000	30 (49.2)	28 (52.8)		
> 10000	14 (23.0)	16 (30.2)		
	3 (4.9)	1 (1.9)		
Medical payment method				
Worker health	12 (19.7)	7 (13.2)	2.433^b^	0.488
Residents of social security	35 (57.4)	35 (66.0)		
At own expense	0 (0)	10 (1.9)		
new rural cooperative medical system	14 (23.0)	10 (18.9)		
Fertility history				
Yes	60 (98.4)	52 (98.1)	0.010^b^	0.92
No	1 (1.6)	1 (1.9)		
FIGO staging				
IIB	31 (50.8)	30 (56.6)	6.488^b^	0.166
IIIA	5 (8.2)	4 (7.5)		
IIIB	8 (13.1)	2 (3.8)		
IIIC	14 (23.0)	17 (32.1)		
IVA	3 (4.9)	0 (0)		
Pathological classification				
Squamous carcinoma	56 (91.8)	47 (88.7)	0.317^b^	0.537
Adenocarcinoma	5 (8.2)	6 (11.3)		
Past medical history				
No	45 (73.8)	36 (67.9)	2.074^b^	0.557
Hypertension	11 (18.0)	8 (15.1)		
Diabetes	1 (1.6)	2 (3.8)		
Heart disease	4 (6.6)	7 (13.2)		
KPS				
70	3 (4.9)	3 (5.7)	0.836^b^	0.405
80	16 (26.2)	15 (28.3)		
90	22 (36.1)	23 (43.4)		
100	20 (32.8)	12 (22.6)		

a t-test; ^b^X^2^ test.

FIGO, Federation International of Gynecology and Obstetrics; KPS, Karnofsky Performance Status.

### Efficacy of radiotherapy

The complete remission (CR) rate was 49.2%, and the partial remission (PR) rate was 44.3% in the MG. The CR rate was 64.2% and the PR rate was 32.1% in the EG. The total effective rates (PR + CR) of the MG and EG were 93.5% and 96.3%, respectively. The CR rate of the EG was slightly higher than that of the MG, although further analysis showed that there was no significant difference in the CR rate between the two groups (*p* > 0.05). The results are described in **Table 4**.

**Table 4:** Radiotherapy efficacy and efficacy rate.

Group	CR (%)	PR (%)	SD (%)	PD (%)	*x^2^*	*p*
MG	30 (49.2)	27 (44.3)	3 (4.9)	1 (1.6)	3.177	0.365
EG	34 (64.2)	17 (32.1)	2 (3.7)	0 (0)		

CR, complete remission; PR, partial remission; SD, stable disease; PD, progression of disease.

### Radiotherapy toxicity, symptoms, and related QOL outcomes

In this study, the main toxic reactions were radioactive gastrointestinal reactions and myelosuppression, and the toxicity levels were 0, I, II, III, and IV. There were significant differences in the incidence of myelosuppression and intestinal reaction between the MG and EG (*p* < 0.05). The MDASI score of the MG was slightly higher than that of the EG (21.64 ± 7.916 vs. 18.53 ± 4.098, *p* < 0.05). In terms of QOL, physical activity, functional status, and overall QOL of the MG were significantly poorer than those of the EG (*p* < 0.05). No other measures showed a significant difference between the groups. The results are described in **Tables 5**–**7**.

**Table 5:** Radiotherapy toxicity.

Group	Number	Grade 0	Grade 1	Grade 2	Grade 3	Grade 4	*x^2^*	*p*
Radiation enteritis (%)								
MG EG	61 53	17 (27.9) 29 (54.7)	24 (39.3) 18 (34.0)	17 (27.9) 5 (9.4)	3 (4.9) 1 (1.9)	0 (0) 0 (0)	11.026	0.012
Diarrhea (%)								
MG EG	61 53	21 (34.4) 33 (62.3)	27 (44.3) 13 (24.5)	8 (13.1) 6 (11.3)	4 (6.6) 1 (1.9)	1 (1.6) 0 (0)	10.141	0.038
Radiocystitis (%)								
MG EG	61 53	45 (73.8) 39 (73.6)	9 (14.8) 5 (9.4)	5 (8.2) 7 (13.2)	2 (3.3) 2 (3.8)	0 (0) 0 (0)	1.350	0.717
Nausea/vomiting (%)								
MG EG	61 53	20 (32.8) 26 (49.6)	20 (32.8) 15 (28.3)	14 (23.0) 10 (18.9)	6 (9.8) 1 (1.9)	1 (1.6) 1 (1.9)	5.199	0.267
Radiodermatitis (%)								
MG EG	61 53	45 (73.8) 41 (77.4)	8 (13.1) 10 (18.9)	7 (11.5) 2 (3.8)	1 (1.6) 0 (0)	0 (0) 0 (0)	3.643	0.303
Myelosuppression (%)								
MG EG	61 53	24 (39.3) 14 (26.4)	23 (27.7) 17 (32.1)	13 (21.3) 12 (22.6)	1 (1.6) 9 (17.0)	0 (0) 1 (1.9)	10.462	0.033

MG, morning radiotherapy group; EG, evening radiotherapy group.

**Table 6:** M.D. Anderson Symptom Inventory scores.

Group	Number	Average score (mean ± standard deviation)	*t*	*p*
MG	61	21.64 ± 7.916	2.576	0.002
EG	53	18.53 ± 4.098		

MG, morning radiotherapy group; EG, evening radiotherapy group.

**Table 7:** Quality of life (QOL) scores.

Dimension	MG (mean ± standard deviation)	EG (mean ± standard deviation)	*t*	*p*
Physical activity	13.11 ± 4.53	14.53 ± 2.55	7.189	0.047
Family status	15.13 ± 5.83	16.34 ± 4.23	1.249	0.214
Emotional status	7.18 ± 3.55	7.49 ± 3.47	0.470	0.639
Functional status	14.03 ± 2.65	15.15 ± 3.10	2.075	0.040
Additional	13.80 ± 5.49	14.64 ± 5.73	0.797	0.427
Total QOL score	63.26 ± 8.68	68.15 ± 10.04	2.789	0.006

MG, morning radiotherapy group; EG, evening radiotherapy group; QOL, quality of life.

## Discussion

### Radiotherapy toxicity

In nature, from simple single-celled organisms to complex mammals and humans, there are certain periodic life activities. Circadian rhythm is a special internal timing mechanism with a 24-hour cycle that is produced by the body, and it can self-regulate and change from day to night (19). The growth of normal human tissues and cells is precisely regulated by the circadian rhythm. Studies have shown that the cell cycle, proliferation, and cell death are closely related to the circadian clock; thus, the disruption of the circadian rhythm is likely involved in cancer development and progression (20). Radiotherapy remains the main treatment for cervical cancer at present (2). Basic research results have confirmed that the sensitivity of different cells to radiation varies significantly depending on its cycle, and each stage of the cell cycle corresponds to different degrees of radiation sensitivity (21, 22). The sensitivity of cells to radiation varies with the cell cycle; therefore, selecting an appropriate radiotherapy time is crucial. Radiotherapy aimed at the sensitive period of tumor cells while avoiding the sensitive period of healthy tissues can achieve the maximum killing effect on cancer cells and minimize damage to healthy cells (22).

In this study, we implemented chronoradiotherapy under the condition of ethical review. For observations of acute radiation adverse reactions, we found that the incidence of radiation enteritis in the morning was higher than that in the evening (above grade II: 32.8% vs. 11.3%, *p* < 0.05). Diarrhea in the MG was more serious than that in the EG, and the diarrhea of grade II and above was significantly more serious in the MG than in the EG (above grade II: 21.3% vs. 13.2%, *p* < 0.05). Additionally, the degree of myelosuppression was more severe in the EG than in the MG (above grade II: 22.9% vs. 41.5%, *p* < 0.05). Chang et al. (23) randomly divided 67 patients into MG (9:00–11:00 AM) and EG (9:00–11:00 PM) groups, and results showed that the incidence of grade III–IV diarrhea in the MG and EG was 12.5% and 6.1%, respectively. In the EG, the incidence of serious hematological toxicity was significantly higher than that in the MG, which is consistent with our results. A systematic Cochrane review in 2018 included two RCTs with a total sample size of 294 patients treated with radiotherapy for cervical cancer (24). Results showed that the incidence of grade I–II diarrhea in cancer patients was lower in the EG than in MG.

Diarrhea caused by radiotherapy in the pelvic region is mainly caused by intestinal crypt cell apoptosis (25). In a study of the intestinal crypt in mice, an obvious circadian rhythm was observed in the number of apoptotic cells in the intestinal crypt during the administration of radiotherapy at different times, which indicated that radiotherapy-induced apoptosis occurs in a time-dependent manner (26, 27). Studies on the effects of radiation therapy on mice have shown that the induction of apoptosis peaks between 9:00 AM and 11:00 AM and troughs between 7:00 PM and 9:00 PM. Therefore, the occurrence of toxic reactions, such as diarrhea and mucositis, is more serious in the morning than in the evening (13). Myelosuppression and hematological toxicity are more serious in the evening after radiotherapy, which may be because proliferation and apoptosis of bone marrow cells exhibit circadian rhythm changes; indeed, apoptosis in the evening group was significantly higher than that in the morning group (28, 29).

### Radiotherapy effect

Before radiotherapy, there were no significant differences in general demographic, disease, or social data between the two groups of patients. In this study, RECIST 1.1 was used to evaluate the efficacy of radiotherapy in patients with cervical cancer. After radiotherapy, the total effective rates (CR + PR) of the MG and EG were similar (93.5% vs. 96.3%, *p* > 0.05). Moreover, no significant differences in treatment response or disease progression were found between the MG and EG.

The results of this study are consistent with the report of Chang et al. (23) on the treatment of patients with cervical cancer by chronoradiotherapy. Chang et al. (23) randomized 67 cervical cancer patients to evaluate the efficacy of radiotherapy delivered using RECIST 1.1 in the morning and evening. Results showed that the effects were similar in the MG and the EG, and the total effective rates (CR + PR) were 100%, which was consistent with the results of our study. However, Guo et al. (30) evaluated the short-term efficacy of chronoradiotherapy using RECIST 1.1 in 25 cervical cancer patients and found that the effective rates of the MG and EG were 61.5% and 80.0%, respectively, which were significantly different. This finding is inconsistent with the results of our study. Possible reasons for this discrepancy are our sample size was too small, affecting the statistical analyses; or the efficacy of chronoradiotherapy was evaluated after the treatment, and the tumors of some patients regressed, which may have affected the evaluation results. Therefore, we plan to follow up patients over a longer period to evaluate long-term efficacy.

### General condition of symptoms and QOL

The results of this study showed that the MDASI score in the MG was significantly higher than that in the EG (21.64 ± 7.916 vs. 18.53 ± 4.098, *p* < 0.05). Cervical cancer patients experience different degrees of symptoms during radiotherapy, including fatigue, nausea, vomiting, diarrhea, and pain (31). This may be because, during radiotherapy for cervical cancer, the deep penetration of radiation and the numerous organs in the pelvic cavity with similar anatomical positions results in normal tissues and organs being affected by radiation, inducing a series of corresponding symptoms and reactions (32). The MDASI scores were slightly higher in the MG than in the EG, which may be attributed to the higher incidence of radiation enteritis and diarrhea in the MG. Most patients experience nausea, vomiting, loss of appetite, pain, fatigue, and other symptoms (2, 33), which seriously affect the daily lives and QOL of patients, which may explain the higher total MDASI score in the MG than in the EG.

In terms of QOL, the total QOL score in the MG (63.26 ± 8.68) was lower than that in the EG (68.15 ± 10.04). In addition, the scores of physical activity and functional activity in the MG were also slightly lower than those in the EG. A previous study (34, 35) on the application of chronoradiation in patients with head and neck cancer found that the QOL score of the EG was higher than that of the MG during the first and second weeks after the start of radiotherapy, although the difference was not significant. The discrepancy between the results of the two studies may be due to the different biological rhythms and time points of radiosensitivity between the two cancer types. Patients with cervical cancer have a higher incidence of gastrointestinal reactions in the morning, whereas head and neck cancer patients have a higher incidence of oral mucosa in the evening. Alternatively, the time points for the QOL life assessments may have been inconsistent between the two studies.

### Limitations

In this study, the sample size was small, and the observation time for efficacy was short. We only examined the short-term efficacy of radiotherapy. Thus, further longitudinal investigations of the long-term efficacy and toxicity of radiotherapy are needed.

## Conclusion

This multicenter randomized controlled trial focused on the short-term efficacy and side effects of chronoradiotherapy in patients with cervical cancer. We verified that the efficacy of radiotherapy was similar irrespective of whether it was administered in the morning or the evening. However, toxicity and side effects differed depending on the time of radiotherapy administration. That is, more severe hematologic toxicity and greater bone marrow suppression were observed in the EG, whereas more severe gastrointestinal toxicity was observed in the MG. Post-radiation assessment revealed that the overall severity of symptoms in the MG was greater than that in the EG; moreover, the QOL of the MG was lower than that of the EG.

## Data availability statement

The original contributions presented in the study are included in the article/supplementary material. Further inquiries can be directed to the corresponding author.

## Ethics statement

The studies involving human participants were reviewed and approved by Medical Ethics Committee of Tianjin Medical University Cancer Institute & Hospital, China. The patients/participants provided their written informed consent to participate in this study. Written informed consent was obtained from the individual(s) for the publication of any potentially identifiable images or data included in this article.

## Author contributions

Study design: WY, QWM. Clinical subject recruitment: ZBZ. Clinical data collection: CXC, LXF, XJ, YR, LXH, ZZL, WCL and LYL. Primary outcome assessor: LJQ and SAM. Data interpretation: All authors. All authors contributed to the article and approved the submitted version.

## Funding

This study was supported by the Basic Scientific Research Fund of Tianjin Medical University (general project 2020KJ144).

## Conflict of interest

The authors declare that the research was conducted in the absence of any commercial or financial relationships that could be construed as a potential conflict of interest.

## Publisher’s note

All claims expressed in this article are solely those of the authors and do not necessarily represent those of their affiliated organizations, or those of the publisher, the editors and the reviewers. Any product that may be evaluated in this article, or claim that may be made by its manufacturer, is not guaranteed or endorsed by the publisher.
